# Targeting phosphoinositide signaling in cancer: relevant techniques to study lipids and novel avenues for therapeutic intervention

**DOI:** 10.3389/fcell.2023.1297355

**Published:** 2023-10-25

**Authors:** Alicia Llorente, Ryan M. Loughran, Brooke M. Emerling

**Affiliations:** Cancer Metabolism and Microenvironment Program, Sanford Burnham Prebys, La Jolla, CA, United States

**Keywords:** phosphoinositide, lipid, metabolism, cancer, signaling, PI5P4K, lipidomics, mass spectometry

## Abstract

Phosphoinositides serve as essential players in numerous biological activities and are critical for overall cellular function. Due to their complex chemical structures, localization, and low abundance, current challenges in the phosphoinositide field include the accurate measurement and identification of specific variants, particularly those with acyl chains. Researchers are intensively developing innovative techniques and approaches to address these challenges and advance our understanding of the impact of phosphoinositide signaling on cellular biology. This article provides an overview of recent advances in the study of phosphoinositides, including mass spectrometry, lipid biosensors, and real-time activity assays using fluorometric sensors. These methodologies have proven instrumental for a comprehensive exploration of the cellular distribution and dynamics of phosphoinositides and have shed light on the growing significance of these lipids in human health and various pathological processes, including cancer. To illustrate the importance of phosphoinositide signaling in disease, this perspective also highlights the role of a family of lipid kinases named phosphatidylinositol 5-phosphate 4-kinases (PI5P4Ks), which have recently emerged as exciting therapeutic targets for cancer treatment. The ongoing exploration of phosphoinositide signaling not only deepens our understanding of cellular biology but also holds promise for novel interventions in cancer therapy.

## 1 Introduction

**FIGURE 1:**
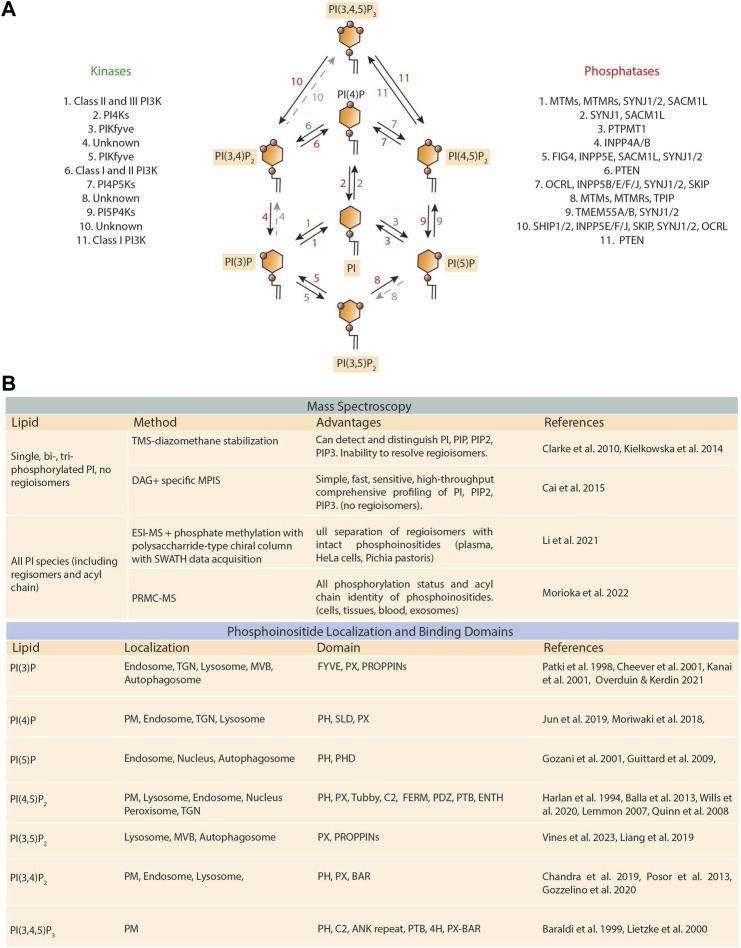
Lipid enzymes that regulate phosphoinositide metabolism and techniques for phosphoinositide measurement and identification. **(A)** Schematic representation displaying the lipid kinases (shown in green) and phosphatases (depicted in red) involved in the generation of the seven phosphoinositide species. Phosphoinositides are formed through the phosphorylation of the third, fourth, and fifth hydroxyl groups on the inositol ring of phosphatidylinositol (PI). The green and red numbers correspond to the lipid kinases and phosphatases that allow each reaction. **(B) *(top)*** Table of phosphoinositide relevant mass spectrometry advancements. TMS-diazomethane, trimethylsilyl diazomethane; DAG + specific MPIS, charged diacylglycerol fragment ion-specific multiple precursor ion scanning; ESI-MS, electrospray ionization mass spectrometry; SWATH, sequential window acquisition of all theoretical mass spectra; PRMC-MS, phosphoinositide regioisomer measurement by chiral column chromatography and mass spectrometry. ***(bottom)*** Localization and binding domains of the seven unique phosphoinositide species. TGN, trans-Golgi network; MVB, multivesicular body; PM, plasma membrane; FYVE, Fab1, YOTB, Vac1, and EEA1; PX, phox homology; PROPPIN, β-propellers that bind phosphoinositides; PH, pleckstrin homology; PHD, plant homeobox domain; SLD, simple-like domain; C2, protein kinase C conserved region 2; FERM, Four-point-one, ezrin, radixin, moesin; PDZ, PSD95, DlgA, Zo-1; PTB, phosphotyrosine binding; ENTH, epsin NH2-terminal homology; BAR, Bin-amphiphysin-Rvs.

The phosphoinositide family of phospholipids plays pivotal roles in nearly all aspects of cellular function. These phospholipids are one of the most functionally versatile membrane lipid families involved in human health and disease (Di Paolo et al., 2006). The base structure of all phosphoinositides contains phosphatidylinositol (PI), which is made up of an inositol head group and two long-chain fatty acids linked to a glycerol backbone. Combinatorial phosphorylation of residues in the PI head group gives rise to seven other PI classes, namely, PI(3)P, PI(4)P, PI(5)P, PI(3,4)P_2_, PI(3,5)P_2_, PI(4,5)P_2_, and PI(3,4,5)P_3_ (Figure 1A). These lipids spatiotemporally control the activities of numerous proteins possessing phosphoinositide-binding motifs and, importantly, these motifs can bind to various PI species with differing affinities to regulate physiological processes in cells.

In addition to the lipids themselves, the importance of phosphoinositide signaling is underscored by the mutations and/or dysregulation of the of PI-metabolizing enzymes, such as kinases, phosphatases, lipases, and acyltransferases, which have been implicated in the pathogenesis of various diseases, including cancer (Burke et al., 2023). Current challenges in the phosphoinositide field include accurately measuring the lipid substrate and reaction products of these important PI enzymes, as well as assessing specific phosphoinositide acyl variants. Here, we will attempt to summarize current techniques to study phosphoinositides and focus on an underappreciated phosphoinositide enzyme family that is emerging as a promising target for several cancer subtypes.

## 2 Advances in phosphoinositide detection techniques

Measuring cellular levels of phospholipids is a continuously developing field, as technical limitations to localization, polarity, and cellular concentrations prove difficult for accurate measurements. Lipidomics, the practice of measuring the different lipid classes in parallel, has proven successful for broad classification and identification of lipid concentrations of several different species. However, the acidic nature of phosphoinositides makes them challenging to measure using these methods. Despite their difficult nature, critical advances in mass spectrometry (MS), lipid biosensors, and sensor-based activity assays have provided an intriguing new outlook for the future of measuring phosphoinositides.

### 2.1 Elucidating regioisomers and acyl chain specificities of phosphoinositides

There are several challenges in investigating the distribution of phosphoinositide species using MS techniques. For a historical review of the utility of MS-based detection of phosphoinositides, we point the reader to an excellent review (Kim et al., 2010). The traditional protocol utilizing extraction, separation, and autoradiograph analysis was sufficient for comparing the relative levels of the eight phosphoinositide classes. Major advancements in absolute quantification of phosphoinositide species (Figure 1B) were initially sparked by the methylation of phosphoinositides using TMS-diazomethane to stabilize the phosphate group and allow for subsequent measuring of the species PI, PIP_2_, and PIP_3_. However, this method did not yet delineate regioisomers (Clark et al., 2011; Kielkowska et al., 2014). Using a similar stabilization method with charged diacylglycerol fragment ion-specific multiple precursor ion scanning (DAG-specific MPIS) allowed for rapid identification of PI, PIP_2_, and PIP_3_ species in tissue extracts and human cell lines with additional information on fatty acyl chain variations, but nevertheless, the limitation of identifying the remaining regioisomers of phosphoinositides still remained (Cai et al., 2015; Cai et al., 2016). Recent advancements combining electrospray ionization MS (ESI-MS) and sequential window acquisition of all theoretical fragment ion mass spectra (SWATH) technology enabled the full picture of phosphoinositide regioisomers in human plasma and cultured HeLa cells (Li and Lammerhofer, 2021). This elegant use of advanced technical deconvolution of MS datapoints using SWATH, coupled with strategic mapping of column elution time of regioisomers, allowed for the untangling of miniscule differences between phosphoinositide regioisomers. Similarly, a newly developed technique to measure regioisomers and acyl chain length termed phosphoinositide regioisomer measurement by chiral column chromatography MS (PRMC-MS) was successful in deciphering phosphoinositides and acyl chain length in cells, blood, and cancerous tissue (Morioka et al., 2022). Intriguingly, this method revealed oncogene induced acyl chain signatures and extracellular phosphoinositide mobilization (Morioka et al., 2022). In addition to furthering our knowledge of the cellular phosphoinositide milieu, the use of mass spectrometry imaging (MSI) can provide a powerful tool for uncovering phosphoinositide distribution in tissue-based applications (Buchberger et al., 2018). For instance, high-resolution matrix-assisted laser desorption/ionization imaging MS (MALDI-MS) has been used to identify several phosphoinositide species accumulating in breast cancer tissues (Kawashima et al., 2013). The application for MSI allows for both uncovering the precise spatial distribution of phosphoinositides in breast cancer tissues compared to normal tissue, as well as identifying altered acyl chain distributions of phosphoinositides that could serve as future biomarkers (Kawashima et al., 2013). Further, recent work utilizing MALDI-MS on histological samples from breast cancer patients revealed distinct differences in phosphoinositide acyl chain distribution in invasive cancer cells compared to normal tissues, which when coupled with gene expression analysis, showed an association with PD-1-related immune checkpoint pathway (Kawashima et al., 2020). This can also be extended to *in vivo* metastasis studies, where metastatic brain lesions, originated from orthotopically implanted human MDA-MB-435 breast cancer cells, were found to contain specific altered phosphoinositide populations compared to surrounding tissues (Roux et al., 2023). Taken together, the rigorous identification of phosphoinositide species, including their variations in acyl chain length, may bring the field forward to identifying these alterations as biomarkers in the cancer setting.

### 2.2 Advances in domain-based lipid biosensor detection of phosphoinositides

Phosphoinositides are critically important for cell signaling processes, as their local concentration at both the plasma membrane and intracellular membranes directs recruitment of effector proteins. To date, the subcellular mapping of phosphoinositides present at each membrane is well-defined and can be found in many excellent reviews (Balla et al., 2009; Falkenburger et al., 2010; Balla, 2013; Burke, 2018; Dickson and Hille, 2019). Here, we will focus on the developing progress of fluorescently encoded biosensors based on phosphoinositide interacting domains. The best characterized domains are the PH-, FYVE-, and PX-domains (Lee et al., 2005; Chandra et al., 2019). The largest family of lipid-binding domains is the pleckstrin homology (PH) domain, although a continuously developing list of lipid-binding domains recognizing phosphoinositides now enables the possibility of identifying all species (Figure 1B) (Varnai and Balla, 1998). Intriguingly, the identification of these domains has resulted in the development of genetically encoded biosensors facilitating the study of phosphoinositides in their native cellular context (Greenwald et al., 2018; Hertel et al., 2020; Hammond et al., 2022; Posor et al., 2022). Successful development of these probes requires high selectivity of target lipid (due to low abundance) and the target lipid alone, sans the presence of helper proteins, must drive localization of the protein domain to the site of activity (Hammond and Balla, 2015; Wills et al., 2018).

The drawback to genetically encoded biosensors is the requirement for expression in the cell of interest, thus complicating the accuracy of quantitation, as the expression level of these proteins can be highly variable. To resolve this issue, new biosensor development encompassing Fluorescence Resonance Energy Transfer (FRET)-based and dimerization-dependent fluorescent protein-based biosensors utilizing PH domains enable density-based measurements of localization with the tagged proteins in live cells (van der Wal et al., 2001; Sato et al., 2003; Hertel et al., 2020). Using the dimerization-dependent biosensor strategy, the generation of a series of phosphoinositide reporters that preserve the native cellular environment and importantly, are spatially targetable, enabled subcellular location-specific monitoring of phosphoinositide dynamics at areas away from the plasma membrane (Hertel et al., 2020). Recent work has combined activity assays and a predictive algorithm to accurately predict full-length protein containing-PH domain binding to phosphoinositides (Singh et al., 2021). A similar investigative effort to characterize the binding profile of the 49 known PX-domain containing proteins has led to a new grouping classification system for proteins containing these domains, which bind to not only PI(3)P, but to other phosphorylated phosphoinositides as well (Chandra et al., 2019). In future studies, fine-tuning the ability to predict protein binding and localization based on their domain interactions with phosphoinositides could provide an enhanced toolbox for drug discovery. Recently, several groups have shown the utility of phosphoinositide binding antibodies, although the stabilization of lipids and permeabilization techniques must be highly considered to preserve the native lipid structure after fixation. Nonetheless, these antibodies provide a powerful visualization tool without the requirement of engineered cell lines. Discovery of new phosphoinositide related functions in the nuclear compartment demonstrate the need for nuclear visualization of phosphoinositides (Shah et al., 2013). Antibody labeling in direct comparison to overexpressed domains shows a separate and distinctive patterning that was only replicated by preparing purified domains fused with eGFP (Irino et al., 2012; Kalasova et al., 2016). As our knowledge of protein-binding domains specific to phosphoinositides grows, so too will our capabilities of using new biosensors to delineate phosphoinositide dynamics and localization spatially and temporally to subcellular compartments.

### 2.3 Functional assays for phosphoinositide drug discovery

With our advancing knowledge of measuring phosphoinositide species through MS and using fluorescent probes to further compartmentalize phosphoinositide dynamics at subcellular membranes, quantitatively measuring enzymatic reactions that interconvert these lipids is equally important for a comprehensive view of phosphoinositide relevance in cellular function. Historically, phosphoinositide kinase activity assays were performed using radioactivity-based assays, thus preventing direct measurement of this process in the native cellular context. The development of solvatochromatic fluorophores in conjunction with protein-based lipid sensors allows for quantification of metabolically linked signaling lipids, such as PI(4,5)P_2_ and PI(3,4,5)P_3._ However, this technology is amenable to any combination of signaling molecules (Sharma et al., 2020). Recently, the development of real-time activity assays using fluorometric sensors allows for quantitative analysis of enzyme kinetics in response to small molecule modulators (Sun et al., 2020). This assay has for the first time provided a modular system, capable of rapidly screening small molecule inhibitors to lipid kinases. Indeed, as the landscape of phosphoinositide localization throughout the cell becomes better defined, and we improve domain binding algorithms, targeting lipid-protein interactions may prove to be an attractive target (Saliba et al., 2015; Singaram et al., 2023).

## 3 Integrating lipid biology knowledge into novel approaches for cancer treatment

The techniques discussed above have collectively shed light on the critical significance of maintaining a precise balance of phosphoinositide levels to ensure optimal cellular functionality. Notably, numerous studies have demonstrated a correlation between alterations in the composition of cellular phosphoinositides and the development and progression of several cancer types (Bunney and Katan, 2010). Central to these alterations are lipid kinases, phosphatases, and phospholipases, making these lipid-modifying enzymes promising candidates for targeted cancer therapy. Here, we will highlight the role of phosphatidylinositol 5-phosphate 4-kinases (PI5P4Ks) in cancer as an example of what we believe to be exciting novel therapeutic targets for cancer treatment.

### 3.1 Role of PI5P4Ks in tumor progression

PI5P4Ks phosphorylate PI(5)P to generate PI(4,5)P_2_. Within healthy cells, PI(4,5)P_2_ serves as a platform to activate various signaling pathways that regulate crucial cellular processes, such as cell growth, proliferation, migration, and apoptosis (Llorente et al., 2023). In the context of cancer, variations in PI(5)P and PI(4,5)P_2_ levels disrupt normal cellular signaling, fostering cancer cell survival and contributing to tumor progression and metastasis (Arora et al., 2022). Deregulated expression of PI5P4Ks has been reported in several cancer types, including leukemia, glioblastoma, soft tissue sarcoma, and prostate and breast cancers (Emerling et al., 2013; Jude et al., 2015; Shin et al., 2019; Ravi et al., 2021; Triscott et al., 2023).

#### 3.1.1 Localization and impact

The PI5P4K family of lipid kinases include three members: PI5P4Kα, PI5P4Kβ, and PI5P4Kγ, with differences in their catalytic activity and localization within the cell. While all three PI5P4K isoforms are primarily found within intracellular membranes, their precise subcellular distributions vary. PI5P4Kα is distributed across lysosomes, autophagosomes, and peroxisomes; PI5P4Kβ primarily resides within the nucleus but may also be detected in autophagosomes, and PI5P4Kγ can be observed in autophagosomes, endomembrane compartments, and the Golgi apparatus (Clarke et al., 2008; Bultsma et al., 2010; Vicinanza et al., 2015; Hu et al., 2018; Lundquist et al., 2018). Additionally, their localization can be influenced by their capacity to heterodimerize (Bultsma et al., 2010; Wang et al., 2010). Interestingly, recent data has shown that PI5P4Ks can also be recruited to the plasma membrane by PI(4,5)P_2_ to inhibit phosphatidylinositol-4-phosphate 5-kinases (PI4P5Ks) as a homeostasis mechanism (Wills et al., 2023). By regulating the balance between PI(5)P and PI(4,5)P_2_, PI5P4Ks modulate cellular signaling, protein activation at specific subcellular locations and protein transport. Furthermore, growing evidence suggests that these kinases possess catalytic-independent roles, which adds a layer of complexity to their functional repertoire (Llorente et al., 2023). Notably, *in vitro* assays have shown a markedly higher kinase activity for PI5P4Kα when compared to PI5P4Kβ, while PI5P4Kγ exhibits the lowest activity (Clarke et al., 2008; Bultsma et al., 2010; Wang et al., 2010). The substantial difference in kinase activity, with PI5P4Kγ being several orders of magnitude lower than the other two kinases, suggests that it potentially plays a more significant role as a scaffolding protein.

#### 3.1.2 Metabolic adaptations through the action of PI5P4Ks in tumor cells

During tumorigenesis, cancer cells typically rely on metabolic reprogramming to adapt to energy and oxidative stresses. The synthetic lethal interaction involving p53, PI5P4Kα, and PI5P4Kβ is a very compelling illustration of this phenomenon (Emerling et al., 2013). The frequent loss of p53 in cancer renders cells more susceptible to oxidative stress. In this context, PI5P4Kα and PI5P4Kβ play critical roles in regulating oxygen consumption, ROS generation, glucose metabolism and AKT signaling to manage such stress, allowing cancer cell survival despite challenging conditions. Other studies support the role of PI5P4Ks in metabolic homeostasis through modulation of insulin signaling, PI3K, AKT, and mTORC pathways, and oxidative stress (Carricaburu et al., 2003; Gupta et al., 2013; Mackey et al., 2014; Lundquist et al., 2018; Wang et al., 2019). It is worth noting that PI5P4Kβ preferentially uses GTP over ATP for PI(4,5)P_2_ synthesis, acting as an intracellular GTP sensor. The GTP-sensing ability of PI5P4Kβ plays a crucial role in both metabolic adaptation and tumor development (Sumita et al., 2016).

PI5P4Ks have also been implicated in autophagy and organelle communication. Loss of PI5P4Ks expression increases autophagosome biogenesis and results in a defect in autophagosome-lysosome fusion and subsequent accumulation of autophagic vesicles (Vicinanza et al., 2015; Lundquist et al., 2018). In addition, PI5P4Kα regulates PI(4,5)P_2_ levels at the peroxisomal membrane to facilitate the lysosome-peroxisome membrane contacts necessary for proper intracellular cholesterol transport (Hu et al., 2018). Moreover, regulation of the peroxisomal PI(4,5)P_2_ pool by the action of PI5P4Kα and PI5P4Kβ is also required for the traffic and peroxisomal oxidation of very long chain fatty acids and consequently for ensuring proper mitochondrial metabolism (Ravi et al., 2021). Consistently, inhibition of these kinases negatively impacts mitochondrial ATP production, disrupting cell energy metabolism (Chen et al., 2021). Together, all these studies demonstrate the ability of PI5P4Ks to maintain the metabolic homeostasis required for cancer cell survival.

Deregulation of stress response pathways, such as autophagy, frequently contributes to drug resistance and tumor progression. Given the implication of PI5P4Ks in energy stress responses and their reported involvement in cellular trafficking (Kamalesh et al., 2017), including membrane receptor recycling (Zheng and Conner, 2018), we speculate that in the context of cancer, PI5P4Ks might play a role in fostering resistance to targeted therapies, especially those involving membrane receptors. Exploring the interplay between PI5P4Ks and resistance pathways would provide valuable insights for enhancing the durability and effectiveness of targeted cancer treatments.

#### 3.1.3 Immunomodulatory roles of PI5P4Ks

Beyond their influence on cancer cell intrinsic processes, PI5P4Ks may also contribute to immune modulation within the tumor microenvironment. Single nucleotide polymorphisms (SNPs) near PIP4K2C (the gene coding for PI5P4Kγ) have been associated with susceptibility to autoimmune diseases (Raychaudhuri et al., 2008; Fung et al., 2009). Interestingly, mice lacking PI5P4Kγ exhibit increased T helper (T_h_) and decreased regulatory T (T_reg_) cell populations, along with heightened proinflammatory cytokine levels, resulting in immune hyperactivation (Shim et al., 2016). Furthermore, there is evidence indicating the necessity of PI5P4Kβ and PI5P4Kγ for T_reg_-mediated immune suppression (Poli et al., 2021). The activity of these PI5P4K isoforms impacts the PI3K, mTORC1 and MAPK signaling pathways, leading to consequential changes in FOXP3 expression that govern T_reg_ reprogramming and functionality. Collectively, these data reveal the significant role of these kinases in immune regulation, underscoring the potential of targeting PI5P4Kγ as a therapeutic strategy to enhance cancer immunotherapy and open new possibilities for cancer treatment.

### 3.2 PI5P4Ks as targets for cancer therapy

**FIGURE 2:**
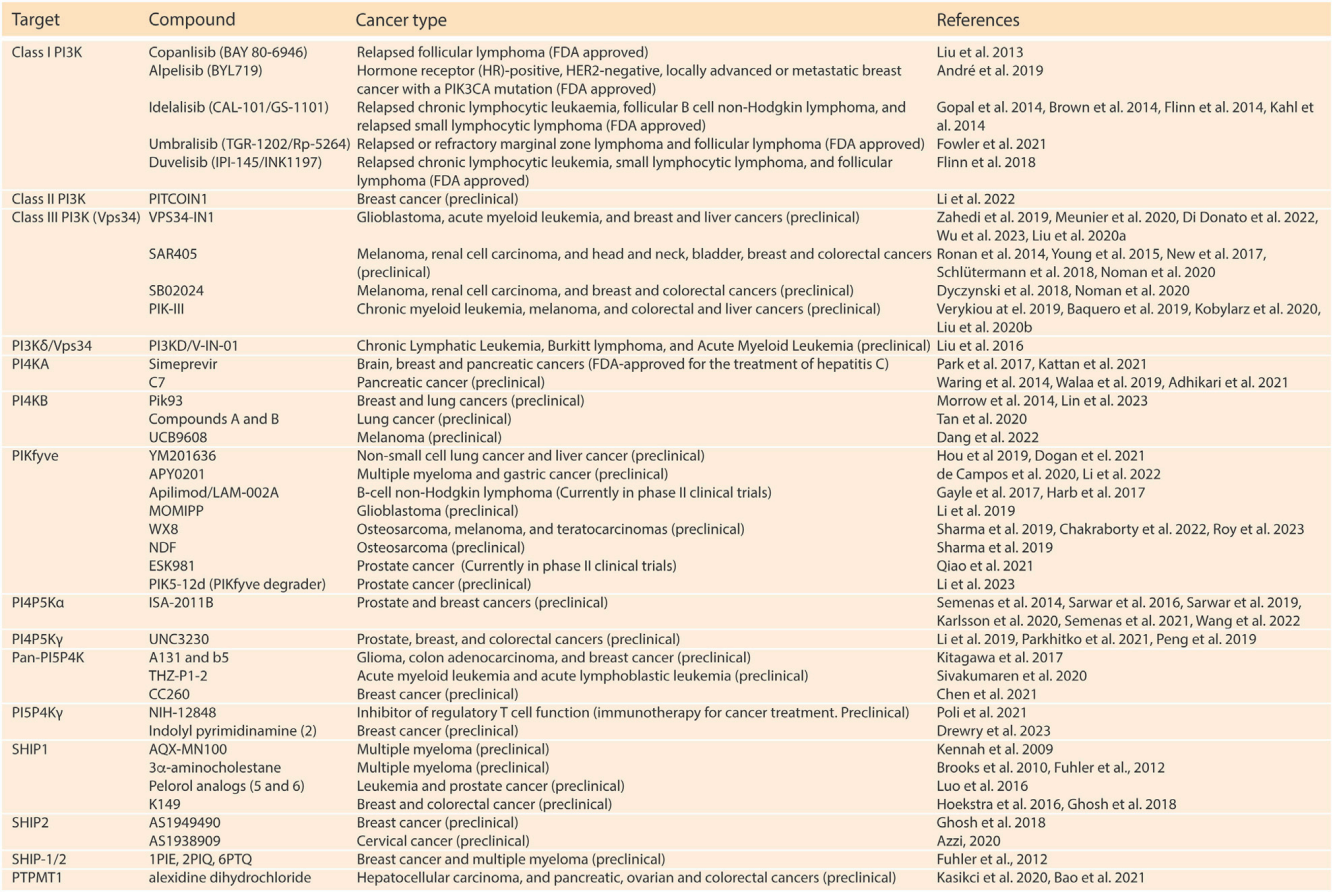
Relevant compounds targeting phosphoinositide kinases and phosphatases for the treatment of cancer. (Harlan et al., 1994; Patki et al., 1998; Baraldi et al., 1999; Lietzke et al., 2000; Cheever et al., 2001; Kanai et al., 2001; Gozani et al., 2003; Quinn, Behe, and Tinker, 2008; Guittard et al., 2009; Kennah et al., 2009; Brooks et al., 2010; Fuhler et al., 2012; Liu et al., 2013; Brown et al., 2014; Flinn et al., 2014; Gopal et al., 2014; Kahl et al., 2014; Morrow et al., 2014; Ronan et al., 2014; Semenas et al., 2014; Waring et al., 2014; Young et al., 2015; Hoekstra et al., 2016; Liu et al., 2016; Luo et al., 2016; Sarwar et al., 2016; Gayle et al., 2017; Harb et al., 2017; Kitagawa et al., 2017; New et al., 2017; Park et al., 2017; Dyczynski et al., 2018; Flinn et al., 2018; Ghosh et al., 2018; Moriwaki et al., 2018; Schlütermann et al., 2018; Li L. et al., 2019; André et al., 2019; Baquero et al., 2019; Li Z. et al., 2019; Chandra et al., 2019; Hou et al., 2019; Jun et al., 2019; Liang et al., 2019; Peng et al., 2019; Sarwar et al., 2019; Sharma et al., 2019; Verykiou et al., 2019; Walaa et al., 2019; Zahedi et al., 2019; Liu F. et al., 2020; Azzi, 2020; Liu et al., 2020b; de Campos et al., 2020; Gozzelino et al., 2020; Karlsson et al., 2020; Kasikci et al., 2020; Kobylarz et al., 2020; Meunier et al., 2020; Noman et al., 2020; Sivakumaren et al., 2020; Tan et al., 2020; Adhikari et al., 2021; Bao et al., 2021; Chen et al., 2021; DoĞan et al., 2021; Fowler et al., 2021; Kattan et al., 2021; Overduin and Kervin, 2021; Parkhitko et al., 2021; Poli et al., 2021; Qiao et al., 2021; Semenas et al., 2021; Li et al., 2022a; Li et al., 2022b; Chakraborty et al., 2022; Dang et al., 2022; Di Donato et al., 2022; Wang et al., 2022; Drewry et al., 2023; Li et al., 2023; Lin et al., 2023; Roy et al., 2023; Wu et al., 2023)

Alterations in phosphoinositide metabolism play a critical role in various human diseases, offering numerous opportunities for therapeutic modulation of the enzymes involved in this process. Currently, significant research efforts have resulted in the development of several compounds targeting lipid kinases and phosphatases in multiple cancer subtypes, with some PI3K inhibitors already having received clinical approval (Figure 2). Further, an increase in the understanding of PI5P4Ks functions have brought to the surface their potential to serve as therapeutic targets for cancer treatment. Here, we are going to provide a short overview of PI5P4K targeting agents that have been developed until now. For an extensive review on the preclinical and clinical development of PI5P4K and other phosphoinositide kinase inhibitors we refer the reader to (Burke et al., 2023).

#### 3.2.1 Small molecule inhibitors

Developing small molecule inhibitors against PI5P4Ks emerges as a promising avenue, allowing precise modulation of PI(5)P and PI(4,5)P_2_ dynamics and its downstream effects. Efforts have been made to develop pan-PI5P4K inhibitors (Kitagawa et al., 2017; Manz et al., 2020; Sivakumaren et al., 2020; Chen et al., 2021; Lima et al., 2022), as well as isoform-specific inhibitors that selectively target PI5P4Kα (Davis et al., 2013; Wortmann et al., 2021; Willems et al., 2023), PI5P4Kβ (Voss et al., 2014), and PI5P4Kγ (Clarke et al., 2015; Al-Ramahi et al., 2017; Boffey et al., 2022; Drewry et al., 2023; Rooney et al., 2023). Despite a dedicated endeavor to enhance the potency and selectivity of these compounds, PI5P4K inhibitors have not yet advanced to the clinical stage.

#### 3.2.2 PI5P4Ks Proteolysis Targeting Chimeras (PROTACs)

Exploring the potential of PI5P4Ks degraders offers a distinct approach, allowing complete abrogation of protein function, including both kinase-dependent and independent functions. Currently, there is active work directed towards the development of PROTACS targeting PI5P4Kα and PI5P4Kβ, while highly potent and selective PI5P4Kγ degraders have already been documented (Ji et al., 2023; Teng et al., 2023). Further, progress in investigating protein-protein interactions, facilitated by techniques like proximity-dependent biotin identification (BioID) (Roux et al., 2012), hold promise in shedding light on the lesser-understood functions of PI5P4Ks that do not rely on their kinase activity, as well as on their currently less-known upstream regulators. Indeed, the efficacy of this approach to identify lipid kinases relevant in the context of cancer has already been demonstrated. In the exploration of KRAS interactions through BioID, researchers successfully identified the type I lipid kinase PIP5K1A (Adhikari and Counter, 2018). Using proximity-based labeling to identify PI5P4K interactors could uncover significant functions beyond phosphoinositide signaling, providing a rationale for the development of molecules that target kinase-dependent and independent functions and shifting the balance from small molecule inhibitors to PROTACs.

## 4 Discussion

Altogether, with our ever-increasing toolbox for phosphoinositide quantification, localization, and turnover, the combination of these technologies in clinically relevant models can potentially provide proof of concept for advancing drug targeting studies of phosphoinositides and their metabolizing enzymes. Further, enhancing the specificity of genetically encoded biosensors will unveil previously undefined pools of phosphoinositides at areas outside the defined phosphoinositide map that is widely accepted, leading to previously unknown protein localizations and involvement in cellular signaling cascades.

Finally, with the wide breadth of phosphoinositide involvement in cellular functions, it is likely to be understated due to our developing, yet limited ability to fully understand their involvement in these processes through measurement. However, the union of these novel technologies paints a bright future for discovery in the field of phosphoinositide biology and, importantly, for the feasibility of targeting phosphoinositide enzymes in human diseases. This is evident with the rising drug discovery efforts of the PI5P4K family of enzymes for cancer treatment, as highlighted here in this perspective.

## Data Availability

The original contributions presented in the study are included in the article/supplementary material, further inquiries can be directed to the corresponding author.
